# Influenza Vaccination of Romanian Medical Students during COVID-19 Times: From Knowledge to Behavior

**DOI:** 10.3390/vaccines12060594

**Published:** 2024-05-30

**Authors:** Bianca Georgiana Enciu, Andreea Marilena Păuna, Carmen Daniela Chivu, Oana Săndulescu, Anna Crispo, Liliana Veronica Diaconescu, Anca Cristina Drăgănescu, Maria-Dorina Crăciun, Daniela Pițigoi, Victoria Aramă

**Affiliations:** 1 Department of Epidemiology, Carol Davila University of Medicine and Pharmacy, 020021 Bucharest, Romania; bianca.milcu@drd.umfcd.ro (B.G.E.); daniela.pitigoi@umfcd.ro (D.P.); 2 National Centre for Communicable Diseases Surveillance and Control, National Institute of Public Health, 050463 Bucharest, Romania; 3 Military Medical Institute, 010919 Bucharest, Romania; 4 Emergency Clinical Hospital for Children “Grigore Alexandrescu”, 011743 Bucharest, Romania; 5 Department of Infectious Diseases I, Carol Davila University of Medicine and Pharmacy, 020021 Bucharest, Romania; 6 National Institute for Infectious Diseases “Prof. Dr. Matei Balș”, 021105 Bucharest, Romania; 7 Academy of Romanian Scientists, 050044 Bucharest, Romania; 8 Epidemiology and Biostatistics Unit, Istituto Nazionale dei Tumori-IRCCS-Fondazione G. Pascale, 80131 Napoli, Italy; 9 Department of Medical Psychology, Faculty of Medicine, Carol Davila University of Medicine and Pharmacy, 020021 Bucharest, Romania; 10 Department of Pediatrics, Carol Davila University of Medicine and Pharmacy, 020021 Bucharest, Romania

**Keywords:** influenza vaccination, vaccine, medical students, vaccine hesitancy

## Abstract

In Romania, influenza vaccination uptake among healthcare workers decreased over time despite access to the vaccine being constantly improved. The aim of this paper is to provide a comparative analysis of the knowledge and attitudes of Dental Medicine and Medicine students towards recommended vaccinations for healthcare workers, focusing on influenza vaccination. A cross-sectional study was conducted during the entire 2021–2022 academic year. Data were collected using 2 electronic questionnaires which were applied to the students from the Faculty of Medicine (*n* = 883) and, respectively, the Faculty of Dental Medicine of the Carol Davila University of Medicine and Pharmacy, Bucharest, Romania. The questionnaires were offered to 1187 students and completed by 911 students (response rate = 77%). Out of these, 85% (*n* = 778) identified the influenza vaccine as recommended; 35% (*n* = 321) reported getting an annual influenza vaccination; and 37% (333) reported getting an influenza vaccination in the previous season. Overall, 45% (*n* = 222) of the respondents who completed the questionnaires from October 2021 to February 2022 reported that they intend to get vaccinated against influenza in the 2021–2022 season and approximately 8% (*n* = 39) reported that they had already been vaccinated. The multivariable analysis showed that the habit of getting annually vaccinated against influenza as well as the knowledge that influenza vaccine is recommended for all healthcare workers were associated with a higher probability of intending to get vaccinated. The current study emphasizes the need to raise awareness among medical students regarding influenza vaccination and to involve medical education institutions, public health authorities, and healthcare facilities in promoting this vaccination among students since the influenza vaccine uptake rate among medical students included in this study was suboptimal.

## 1. Introduction

Influenza vaccination represents the most effective protective measure against the medical and economic impact caused by influenza infections and their related complications [1]. Healthcare workers play an important role in the management of seasonal influenza epidemics and have a higher risk of infection. A meta-analysis published in 2011 showed that healthcare workers display a higher frequency of asymptomatic infection compared to the general population, which highlights their higher risk of exposure to influenza viruses [2]. The same meta-analysis highlighted the fact that the odds of symptomatic infection are lower, with possible explanations for this situation being previous exposure (natural or through vaccination) [2]. However, healthcare workers continue to present an important burden of influenza infections and represent an important link in the epidemiological process [3]. In case of influenza outbreaks with nosocomial transmission, 20–50% of the entire medical staff may be affected and may further transmit the infection to their patients, who are at risk of developing complications [4].

In addition, healthcare workers play an important role in achieving increased influenza vaccine coverage at the national level. They are a role model and an important source of information regarding influenza vaccination for their patients [5].

In Romania, vaccination against influenza has been recommended for all high-risk groups, including healthcare workers, since 1977, according to the European Commission Decision, the Ministry of Health, ensuring that influenza vaccines are free for healthcare workers [6,7,8,9]. However, the publicly available data regarding influenza vaccine uptake among healthcare workers (HCWs) within the National Vaccination Program showed a decreasing trend over time even if the access to influenza vaccines for at-risk groups has been constantly improved [8,9]. The vaccination uptake among HCWs decreased from 54.7% in the 2013–2014 season (2.7% in the overall population) to 29.4% in the 2014–2015 season (2.5% in the overall population). During the following seasons, an increasing trend was recorded with a vaccination uptake among HCWs of 30.2% in 2015–2016 (3.2% in the overall population), 31.0% in 2016–2017 (2.5% in the overall population), 34.6% in 2017–2018 (5.2% in the overall population), 39.7% in 2019–2020 (7.9% in the overall population), and 45.9% in 2020–2021 (7.9% in the overall population). Starting from 2021–2022, the vaccination uptake again recorded a decreasing trend with a vaccination uptake of 21.5% in 2021–2022 (8.0% in the overall population) and 8.0% in 2022–2023 (8.0% in the overall population). No data were available regarding the influenza vaccine uptake among Romanian HCWs in the 2018–2019 season [8,10].

COVID-19 and hepatitis B vaccinations are also recommended for all healthcare workers according to the legal framework [11,12]. Moreover, starting on 1 December 2023, pneumococcal vaccination has also become recommended for all healthcare workers [9]. No specific recommendations regarding HCWs’ vaccination against varicella, pertussis, tetanus, diphtheria, poliomyelitis, and meningococcus exist in Romania [13], apart from those available for the general adult population since December 2023, i.e., diphtheria, tetanus, and pertussis boosters every 10 years [9].

Given the fact that both Dental Medicine and Medicine students are exposed to influenza and other preventable diseases during their medical practice, as well as the fact that the knowledge, attitudes, and practices towards vaccination are built during medical studies, the aim of this paper is to provide a comparative analysis between Dental Medicine and Medicine students towards influenza vaccination, and by comparison with other vaccinations recommended in Romania for all healthcare workers.

## 2. Materials and Methods

### 2.1. Study Design

A cross-sectional study was conducted, using two electronic questionnaires aiming to explore the knowledge and attitude regarding the measures recommended for reducing occupational biological risk. The first questionnaire was designed for students from the Faculty of Medicine (6th year of study) while the second was dedicated to those from the Faculty of Dental Medicine (4th year of study), Carol Davila University of Medicine and Pharmacy. The questions used were similar for both faculties, pertaining to demographic data (age, sex, year of study, faculty), data related to the medical practice (if they are involved in clinical work and the timespan since they started clinical work), data regarding the knowledge and attitude towards vaccination (if they are aware of the vaccines recommended; if they are vaccinated against influenza, COVID-19, and hepatitis B; if they intend to get vaccinated; and the main sources of information regarding vaccination), data regarding the knowledge and attitude towards standard precautions and the correct behavior after occupational exposure to blood. The questionnaires were created in Google Forms, and all information was gathered in the form of an Excel sheet.

### 2.2. Study Period

Data were collected during the entire 2021–2022 academic year, from 7 October 2021 to 31 May 2022. All students from the Romanian teaching module undergoing the Epidemiology module in their academic training program were invited to complete the online questionnaire at the beginning of the first course of Epidemiology. The Epidemiology module provides students with specific information (regarding infectious diseases, preventive measures, and vaccination), and it was important to evaluate the students’ knowledge and vaccination intention up to this point.

### 2.3. Statistical Analysis

For this paper, we performed a descriptive and comparative analysis of demographic data, data related to the medical practice and the level of knowledge regarding the vaccines recommended to all healthcare workers (influenza vaccine, COVID-19 vaccine, hepatitis B vaccine for which there are clear recommendations, but also other vaccines such as the diphtheria–tetanus–pertussis vaccine, measles–mumps–rubella vaccine, and varicella, meningococcal, and hepatitis A vaccines), and data related to the uptake of influenza, COVID-19, and hepatitis B vaccines [7,8,9,11,12,13]. In addition, we assessed the main sources of information regarding vaccination, the level of awareness about the importance of influenza vaccination, and the intention to get vaccinated against influenza in the 2021–2022 season.

For categorical variables, frequencies and percentages were reported, and for continuous ones, median and interquartile ranges (IQR) were calculated. For comparing the differences between groups, Pearson’s Chi-squared test and Fisher’s exact test were used, with *p*-values of less than 0.05 being considered statistically significant.

In order to identify the factors associated with a higher intention to get vaccinated against influenza, both univariable and multivariable analyses were conducted taking into account different exposures.

For the univariable analysis, odds ratios (OR) and 95% corresponding intervals (95%CI) were estimated. The outcomes considered were as follows: being vaccinated in the 2020–2021 season and being vaccinated or intending to get vaccinated in the 2021–2022 season. The exposures taken into account in the univariable models were as follows: being a student at the Faculty of Medicine, being of female sex, having involvement in clinical work, having knowledge regarding the vaccines recommended for healthcare workers (influenza, COVID-19, and hepatitis B), getting vaccinated annually against influenza, being vaccinated against COVID-19 and hepatitis B, as well as having knowledge of the main sources of information regarding vaccination (medical literature, official bulletins, general practitioners, media, and healthcare facility where they are practicing). In addition, odds ratios (OR) and corresponding 95% confidence intervals (CI) were estimated using an unconditional multivariable logistic regression model [14]. Two models were fitted for this purpose: to associate the habit of getting vaccinated against influenza (in the 2020–2021 and 2021–2022 seasons, respectively) with sex; knowledge regarding the recommendation of influenza, COVID-19, and hepatitis B vaccinations for all healthcare workers; the habit of getting annually vaccinated against influenza; the vaccination status against COVID-19 and hepatitis B; as well as awareness of the main sources of information. The models were adjusted by sex, age (continuous), faculty (Dental Medicine, Medicine), year of study (4th, 6th), and period of practice (<1 year, 1–3 years, >3 years). The descriptive and univariable analyses were carried out in RStudio version 2023.12.0 while the multivariable analysis was carried out using STATA software 16.1 version (StataCorp LLC, College Station, TX, USA).

### 2.4. Ethical Considerations

Participation was voluntary, and data were processed in accordance with data protection legislation. This study was conducted in compliance with the students’ evaluation rules of the Carol Davila University of Medicine and Pharmacy, Bucharest, being included and approved in the internal activity plan for the 2021–2022 academic year of the Department of Epidemiology. Approval from the ethics committee of the Carol Davila University of Medicine and Pharmacy, Bucharest, was obtained to communicate the results in the form of scientific articles and presentations (approval number—9206).

## 3. Results

### 3.1. The Study Population

The questionnaire was distributed to 1187 students and was completed by 911 students (response rate 77%), with a median age of 24 years, and an IQR of 23–24 years, the majority being female (*n* = 651; 71%), which is consistent with the demographic pattern of the healthcare sector in Romania. Out of them, 657 (72%) were 6th-year students at the Faculty of Medicine and 254 (28%) were 4th-year students at the Faculty of Dental Medicine. The demographic characteristics of the respondents are presented in Table 1.

### 3.2. Clinical Practice

Three hundred and fifty-two respondents (39%) reported doing clinical practice or volunteering in a medical facility; most of them were students at the Faculty of Dental Medicine (*n* = 194; 55%). Moreover, 76% of the students from the Faculty of Dental Medicine reported being involved in clinical practice or volunteering, compared to 24% (*n* = 158) of the students from the Faculty of Medicine (*p* < 0.001). Regarding the timespan, since they had started clinical work, most students from the Faculty of Medicine (57; 36%) reported doing medical practice for more than 3 years, while 89 (46%) of the Dental Medicine students reported being involved in dental practice for 1 to 3 years. The differences between the two groups of students are presented in Table 2.

### 3.3. Knowledge Regarding Vaccination

More than 90% of the respondents identified COVID-19 and hepatitis B vaccines as recommended for healthcare workers, with the difference between groups being statistically significant for the COVID-19 vaccine. However, 86% (*n* = 778) identified the influenza vaccine as recommended, with the difference between groups being statistically significant. Overall, 74% of the respondents identified the diphtheria–tetanus–pertussis containing vaccine as recommended for healthcare workers, with a higher percentage of Medicine students recognizing this vaccine as recommended. In addition, we noticed that the students from the Faculty of Medicine were more aware of the importance of any vaccination if the epidemiological situation required it (*p* = 0.008). The results are presented in Table 3.

### 3.4. Source of Information Regarding Vaccination

The respondents reported the main sources of information regarding vaccinations in the medical literature (*n* = 458; 50%), followed by the official bulletins issued by the public health authorities (*n* = 148; 16%). Only 1% of the respondents (10) reported receiving information about vaccination from the staff of the healthcare facility where they were conducting medical practice (Table 4).

### 3.5. Influenza Vaccination Uptake

Overall, 35% (321) of respondents reported getting annual influenza vaccination and 37% (*n* = 333) reported getting the influenza vaccination in the 2020–2021 season, with the differences being statistically significant between the two groups of students. By comparison, 97% (*n* = 880) of respondents reported being vaccinated against COVID-19 and 62% (*n* = 569) knew that they had been vaccinated against hepatitis B (Table 5).

### 3.6. Reasons for Not Getting Vaccinated against Influenza

The main reason reported for not getting vaccinated against influenza was the fact that they did not consider it to be necessary as they had supposedly never been infected with influenza viruses (*n* = 113; 37%). A higher percentage of students at the Faculty of Dental Medicine (*n* = 53; 27%) reported this reason compared to 15% (*n* = 60) of students at the Faculty of Medicine (*p* < 0.001). Other respondents reported the unavailability of the influenza vaccine as a reason for not getting vaccinated (*n* = 112; 37%). By comparison, none of the respondents reported the unavailability of the COVID-19 vaccine as a reason for not getting vaccinated against COVID-19.

### 3.7. Awareness Regarding the Benefits of Influenza and COVID-19 Vaccinations

An important proportion of students recognized influenza vaccination as the most effective intervention for influenza prophylaxis (*n* = 886; 97%). Almost all (*n* = 653; 99%) of the students from the Faculty of Medicine identified this method of prophylaxis as the most important, compared to 92% (*n* = 233) of the students from the Faculty of Dental Medicine (*p* < 0.001).

Most of the respondents were aware of the importance of influenza and COVID-19 vaccinations for healthcare workers. Thus, 50% (453) recognized the importance of these vaccinations in protecting themselves, their families, and patients and in preventing professional absenteeism. Only 12 respondents (1%) considered that these vaccinations are not important for medical workers. One respondent reported not considering it important that healthcare workers are vaccinated against influenza and COVID-19, while also recognizing some of the benefits.

### 3.8. Assessment of the Intention to Get Vaccinated against Influenza

A total of 45% (*n* = 222) of the respondents who completed the questionnaire from October 2021 to February 2022 reported that they intend to get vaccinated against influenza in the 2021–2022 season, accounting for 29% of the Dental Medicine students and 49% of the Medicine students *(p* < 0.001). Approximately 8% (*n* = 39) reported that they had already been vaccinated at the time of questionnaire completion. The results are presented in Table 6.

### 3.9. Factors Associated with a Higher Intention to Get Vaccinated

#### 3.9.1. Univariable Analysis

The univariable analysis showed that the habit of getting vaccinated annually against influenza, the knowledge that influenza and COVID-19 vaccines are recommended for all healthcare workers, being vaccinated against COVID-19, and using the medical literature as the main source of information regarding vaccination were associated with a higher probability of intending to get vaccinated in the 2020–2021 season and 2021–2022, respectively (Table 7).

#### 3.9.2. Multivariable Analysis

In the multivariable analysis model, after adjusting for potential confounders such as the faculty (Medicine, Dental Medicine), sex (male, female), year of study (4th or 6th), timespan since they had been practicing (<1 year, 1–3 years, >3 years) and age (continuous), the habit of getting vaccinated annually against influenza as well as the knowledge that the influenza vaccine is recommended for all healthcare workers remained significantly associated with the intention to get vaccinated in both seasons (Table 8).

## 4. Discussion

All people working in the healthcare system and carrying out activities involving contact with patients, including students, are at risk of acquiring various infectious diseases, especially respiratory ones such as influenza and COVID-19. They can also transmit respiratory pathogens to their patients or families.

The healthcare workers from dental services are exposed during the activities involving close contact with the patient as well as during the activities involving aerosol-generating maneuvers. By knowing and following the recommended measures to limit the spread of these respiratory pathogens (e.g., vaccination, standard and specific precautions, disinfection, and sterilization protocols, along with management of indoor airflow), the risk of acquiring such an infection, as well as the risk of transmitting a respiratory pathogen to the family or patients decreases [15]. In addition, HCW vaccination can reduce the occurrence of illness and professional absenteeism, which could lower the costs of healthcare services because of lost productivity. Furthermore, some studies showed that patients and their families consider vaccinated HCWs to be the most trustful source of information regarding vaccinations [16].

Despite recommendations, HCWs’ adherence to routine vaccination schemes is often suboptimal. For example, the influenza vaccination coverage among HCWs in the European Union in 2020–2021 had a median of 52% (range: 16–71%) [17]. The decision to get vaccinated is influenced by many factors such as the underestimation of the risk or severity of the disease, the limited access to vaccination, as well as access to information regarding the safety and effectiveness of the vaccines [16].

In this paper, a comparative analysis between Dental Medicine and Medicine students regarding recommended vaccinations for healthcare workers was provided, focusing on influenza vaccination, in order to develop a set of recommendations aimed at improving students’ adherence to influenza vaccination.

The current study reflected that there is an interest in influenza vaccination among medical students, with a high proportion of respondents identifying influenza vaccination as the most effective method of influenza prophylaxis and recognizing the importance of influenza vaccination in the individual protection, of those around and in the prevention of professional absenteeism. These findings are in accordance with those from other studies. For example, a study carried out in Italy between June and July 2020 demonstrated that medical students are more willing to get vaccinated against influenza compared to other categories of students, which indicates that the self-perception of the risk of contracting influenza is associated with higher adherence to vaccination [18]. Moreover, another study showed that the COVID-19 pandemic was a major factor driving HCWs’ adherence to vaccination, to protect families and patients [19]. A multi-center, cross-sectional study involving more than 3000 participants showed that the willingness to avoid the spread of influenza to family members, patients, or the population was strongly associated with the probability of getting the influenza vaccination the following year [20]. Other studies also reported self-protection, patient protection, and the protection of family and friends as the most common reasons for getting vaccinated against influenza [21,22].

However, the proportion of respondents in our study who knew that the influenza vaccine is recommended for all healthcare workers was lower than the proportion of respondents knowing that the COVID-19 vaccine or the hepatitis B vaccine is recommended for healthcare workers. The intense communication campaigns regarding COVID-19 vaccination conducted, especially immediately after the vaccination campaign was launched, with messages and educational events designed for medical students, as well as HCW prioritization for the COVID-19 vaccination and the involvement of medical students in the management of the COVID-19 pandemic (volunteering in healthcare facilities, public health authorities, vaccination centers) were associated with a greater interest of the respondents regarding this vaccination compared to the influenza vaccination. The positive attitude towards COVID-19 vaccination compared to influenza vaccination was also reported in a Cypriot study published in 2022, where it was explained by the increased risk perception of COVID-19, the low-risk perception of influenza, and the publicity of the pandemic [23].

The data presented above suggest the need to develop educational campaigns targeting medical students regarding the burden of influenza and the benefits of influenza vaccination, but also the need to involve more medical students in the epidemiological management of infectious diseases, especially because of the lower percentage of medical students reporting doing medical practice or volunteering in a healthcare facility. In addition, healthcare facilities should promote vaccination among students, medical residents, or volunteers and should facilitate access to vaccination also for these categories, because in the 2020–2021 season, influenza vaccine uptake among medical students included in the study was 37%, which was lower than the influenza vaccination uptake recorded among healthcare workers in the same season [8,10]. A reason for this low vaccine uptake could be the unavailability of influenza vaccine, with this reason being supported by the results of another study including 332 medical students and residents from Romania, showing vaccine accessibility as one of the main barriers to vaccination [4], as prior to the 2023–2024 influenza season, the Ministry of Health used to provide influenza vaccines for healthcare workers within the National Vaccination Program based on the requests sent by the healthcare facilities. These vaccines were mainly used for vaccinating contractual healthcare workers whose number was known. Having clear evidence of medical students requiring vaccines is very difficult, as they are moving from hospital to hospital for different modules of limited duration. To increase the access of high-risk groups to influenza vaccination, starting with the 2023–2024 influenza season, the vaccine is reimbursed [8,9]. However, supplementary lessons should be learned from the COVID-19 vaccination and should be used for improving the uptake of influenza vaccines among HCWs as the COVID-19 vaccination uptake was high among the study participants, similar to that recorded at the national level among healthcare workers [24]. For example, organizing vaccination centers within hospitals or medical academic institutions could facilitate the access of students and HCWs to vaccination, in addition to the reimbursement policies. Even if the COVID-19 vaccine uptake was high among the study participants, the multivariable analysis did not show a significant association between COVID-19 vaccination and the intention to get vaccinated against influenza.

The habit of getting vaccinated against influenza as well as the knowledge that the influenza vaccine is recommended for all healthcare workers remained significantly associated in our study with a higher intention to accept influenza vaccination both in the univariable and multivariable analysis, strengthening the recommendation of raising awareness among medical students about this vaccination. This positive relationship between the intention to get vaccinated and actually getting vaccinated is also translated into a bidirectional effect; specifically, having been previously vaccinated against influenza is an important driver of future vaccination and of positive attitudes toward influenza vaccination, as has also been shown in a recent meta-analysis [25]. The need to intensify the educational process of medical students regarding vaccination was identified even before the COVID-19 pandemic in an important international multicenter study published in July 2020 [26].

In addition, increased involvement of higher education institutions in the training of students on vaccination should be promoted as early as possible, preferably in the first 2 years (preclinical years), given the fact that once the students begin clinical activities in hospitals, they share the same risks to themselves and to their patients as practicing clinicians [27]. Furthermore, there is a recognition that attitudes and biases are formed early in education. Moreover, students’ confidence in the information about influenza vaccination provided by qualified healthcare providers was highlighted in studies conducted among international students [28].

Looking at the differences between the two groups of students, we can notice the fact that a higher proportion of the Medicine students knew that the influenza vaccine is recommended for healthcare workers, and a higher vaccination rate was recorded among them compared with the Dental Medicine students. A Swiss study including 1111 dental healthcare workers reported that only 17.4% of participants received a yearly vaccination against seasonal influenza, these results being in accordance with our study results [29]. By comparison, only 20% of Dental Medicine students reported getting an influenza vaccine annually in our study. The percentage of Dental Medicine students intending to get vaccinated against influenza in the 2021–2022 season is similar to that reported in a Greek study [30].

Although the present study focused on influenza vaccination, other vaccines recommended for HCWs were also evaluated. The COVID-19 vaccine uptake was high among both groups and similar to that reported in the literature, including in studies conducted among HCWs from Romania [4,24,31]. Likewise, it was highlighted that awareness of the risk of contracting the hepatitis B virus and the possibility of chronic evolution of this infection were also associated with a greater interest of the respondents regarding hepatitis B vaccination. However, the percentage of Dental Medicine students knowing their vaccination status against hepatitis B was lower compared to those from Medicine [29].

With regard to the sources of information regarding vaccination, a higher percentage of Dental Medicine students (29%) reported as the main source of information regarding vaccination the courses from the faculty, and this result could be explained by the existence of a prevention course in the Dental Medicine curriculum.

The data presented above highlight the need to develop tailored interventions for medical students to increase adherence to influenza vaccination. According to a meta-analysis published in 2024 including 48 articles on influenza vaccination, multi-component interventions have a greater benefit on HCWs’ adherence to vaccination than mono-component interventions [16], so the reimbursement of the vaccine which addresses the unavailability of the vaccine should be combined with other interventions such as educational interventions given the fact that our study revealed a significant association between the knowledge that the influenza vaccine is recommended for all healthcare workers and the intention to accept influenza vaccination. Another review published in 2022 highlighted the fact that offering the influenza vaccine for free and increasing access to vaccination using convenient locations and hours for this activity were the most important components of an effective vaccination program for university students [32].

### 4.1. Strengths and Limitations

The strength of this study is represented by the fact that it provides an image of the particularities of two main medical branches, Medicine and Dental Medicine, in terms of the perception of influenza vaccination, underlining the need for designing campaigns or courses to raise awareness of the importance of influenza vaccination for students at the Faculty of Dental Medicine while for students at the Faculty of Medicine, it is important to introduce prevention courses in the preclinical years. In addition, according to our knowledge, there is scarce evidence regarding the perception of Dental Medicine students regarding vaccination in general, and influenza vaccination in particular.

The limitations of this study are related to the fact that only students from a single year of study (6th and 4th, respectively) from a single university were included; an extended analysis with data from several medical universities from the country could provide a better picture of the topic addressed. In addition, the present research did not consider the medical conditions or family history of the respondents (e.g., if they were infected with SARS-CoV-2 or if someone in the family had the infection). These aspects could have influenced the attitude towards vaccination.

### 4.2. Directions for Future Research

This study highlights the need for a deeper assessment of medical students’ level of knowledge about influenza (mode of transmission, complications, prevention, and control measures) and perception regarding influenza vaccination in the post-pandemic period. Future research is also important to assess the impact of the new strategies of providing the influenza vaccine for at-risk groups on vaccine uptake among medical students.

## 5. Conclusions

The present study emphasizes the need to raise awareness among medical students regarding influenza vaccination and to involve medical education institutions, public health authorities, and healthcare facilities in promoting this vaccination among students since only a little over a third of respondents reported receiving the influenza vaccination. The interventions should be tailored to every group’s needs.

The knowledge regarding the influenza vaccine recommendation for HCWs was significantly associated with the intention to get vaccinated, an observation that underlines the importance of topics such as vaccination and vaccine recommendation in the study curriculum.

## Figures and Tables

**Table 1:** Demographic characteristics of respondents included in this study, by faculty, at the Carol Davila University of Medicine and Pharmacy, Bucharest, Romania, 2021–2022.

Characteristics	Total *n* = 911 ^1^	Dental Medicine *n* = 254 ^1^	Medicine *n* = 657 ^1^
Sex			
Female	651 (71)	186 (73)	465 (71)
Male	260 (29)	68 (27)	192 (29)
Age (years)	24 (23, 24)	22 (22, 23)	24 (24, 25)
Age groups (years)			
20–24	710 (78)	244 (96)	466 (71)
25–29	190 (21)	7 (3)	183 (28)
30+	11 (1)	3(1)	8 (1)

^1^ *n* (%); median (IQR).

**Table 2:** Distribution of respondents included in the study, according to faculty and time period since they started medical practice in a healthcare facility, Carol Davila University of Medicine and Pharmacy, Bucharest, Romania, 2021–2022.

Time Period	Total *n* = 352 ^1^	Dental Medicine *n* = 194 ^1^	Medicine *n* = 158 ^1^	*p*-Value ^2^
				<0.001
<1 year	129 (37)	74 (38)	55 (35)	
1–3 years	135 (38)	89 (46)	46 (29)	
>3 years	88 (25)	31 (16)	57 (36)	

^1^ *n* (%). ^2^ Pearson’s Chi-squared test.

**Table 3:** The distribution of respondents included in this study, according to the faculty and the level of knowledge regarding the vaccines recommended for healthcare workers, Carol Davila University of Medicine and Pharmacy, Bucharest, Romania, 2021–2022.

Vaccine	Total *n* = 911 ^1^	Dental Medicine *n* = 254 ^1^	Medicine *n* = 657 ^1^	*p*-Value ^2^
***Influenza***				<0.001
Yes	778 (86)	182 (72)	596 (91)	
No	85 (9)	50 (20)	35 (5)	
Don’t know	48 (5)	22 (8)	26 (4)	
***COVID-19***				0.006
Yes	860 (94)	230 (91)	630 (96)	
No	36 (4)	16 (6)	20 (3)	
Don’t know	15 (2)	8 (3)	7 (1)	
***Hepatitis B***				0.093
Yes	875 (96)	241 (95)	634 (96)	
No	9 (1)	1 (0)	8 (1)	
Don’t know	27 (3)	12 (5)	15 (3)	
***Diphtheria–tetanus–pertussis***				0.008
Yes	671 (74)	184 (72)	487 (74)	
No	90 (10)	16 (7)	74 (11)	
Don’t know	150 (16)	54 (21)	96 (15)	
***Any vaccine if the epidemiological situation requires it***				0.006
Yes	805 (88)	211 (83)	594 (90)	
No	38 (4)	17 (7)	21 (3)	
Don’t know	68 (8)	26 (10)	42 (7)	
***Varicella***				0.100
Yes	466 (51)	139 (55)	327 (50)	
No	236 (26)	53 (21)	183 (28)	
Don’t know	209 (23)	62 (24)	147 (22)	
***Hepatitis A***				<0.001
Yes	411 (45)	162 (64)	249 (38)	
No	305 (34)	44 (17)	261 (40)	
Don’t know	195 (21)	48 (19)	147 (22)	
***Measles–mumps–rubella***				0.400
Yes	677 (74)	189 (75)	488 (74)	
No	122 (14)	29 (11)	93 (14)	
Don’t know	112 (12)	36 (14)	76 (12)	
***Meningococcal***				<0.001
Yes	438 (48)	140 (55)	298 (45)	
No	195 (21)	33 (13)	162 (25)	
Don’t know	278 (31)	81 (32)	197 (30)	

^1^ *n* (%). ^2^ Pearson’s Chi-squared test; Fisher’s exact test.

**Table 4:** The main sources of information regarding vaccination, Carol Davila University of Medicine and Pharmacy, Bucharest, Romania, 2021–2022.

Source of Information Regarding Vaccination	Total *n* = 911 ^1^	Dental Medicine *n* = 254 ^1^	Medicine *n* = 657 ^1^
Medical literature	458 (50)	51 (20)	407 (62)
Official bulletins issued by public health authorities	148 (16)	43 (17)	105 (16)
Courses from the faculty	73 (8)	73 (29)	0 (0)
Infectious diseases specialist or medical epidemiologist	71 (8)	14 (6)	57 (8)
Internet/social media	50 (6)	20 (8)	30 (5)
General practitioner	44 (5)	35 (14)	9 (2)
Healthcare facility when I am doing the medical practice	31 (4)	4 (1)	27 (4)
Colleagues	13 (1)	5 (2)	8 (1)
Mass-media (radio, TV, newspapers, news)	13 (1)	6 (2)	7 (1)
I did not receive any information regarding vaccines	10 (1)	3 (1)	7 (1)

^1^ *n* (%).

**Table 5:** The distribution of respondents according to the self-reported vaccination status against influenza, COVID-19, and hepatitis B by faculty, Carol Davila University of Medicine and Pharmacy, Bucharest, Romania, 2021–2022.

Questions and Answers	Total *n* = 911 ^1^	Dental Medicine *n* = 254 ^1^	Medicine *n* = 657 ^1^	*p*-Value ^2^
***Do you use to get the influenza vaccine annually?***				<0.001
Yes	321 (35)	50 (20)	271 (41)	
No	590 (65)	204 (80)	386 (59)	
***Did you get vaccinated against influenza in the past season (2020–2021)?***				<0.001
Yes	333 (37)	55 (22)	278 (42)	
No	578 (63)	199 (78)	379 (58)	
***Are you vaccinated against COVID-19?***				<0.001
Yes	880 (97)	236 (93)	644 (98)	
No	31 (3)	18 (7)	13 (2)	
***Are you vaccinated against hepatitis B?***				<0.001
Yes	569 (63)	110 (43)	459 (70)	
No	210 (23)	80 (32)	130 (20)	
Don’t know	132 (14)	64 (25)	68 (10)	

^1^ *n* (%). ^2^ Pearson’s Chi-squared test.

**Table 6:** The distribution of respondents who completed the questionnaires from October 2021 to February 2022 according to the intention to get vaccinated against influenza in the 2021–2022 season, by faculty, Carol Davila University of Medicine and Pharmacy, Bucharest, Romania, 2021–2022.

*Questions and Answers*	Total *n* = 496 ^1^	Dental Medicine *n* = 106 ^1^	Medicine *n* = 390 ^1^	*p*-Value ^2^
***Are you planning to get vaccinated in the 2021–2022 season?***				<0.001
Yes	222 (45)	31 (29)	191 (49)	
I am already vaccinated	39 (8)	5 (5)	34 (9)	
No	101 (20)	33 (31)	68 (17)	
I don’t know	134 (27)	37 (35)	97 (25)	

^1^ *n* (%). ^2^ Pearson’s Chi-squared test.

**Table 7:** Univariable analysis of different exposures on the intention to get vaccinated against influenza in 2020–2021 and 2021–2022 seasons, Carol Davila University of Medicine and Pharmacy, Bucharest, Romania, 2021–2022.

	Vaccination Intention 2020–2021		Vaccination Intention 2021–2022	
	OR (95% CI)	*p*-Value	OR (95% CI)	*p*-Value
Medicine	2.8 (2.0–3.8)	<0.001	2.7 (1.9–3.8)	<0.001
Female sex	1.2 (0.9–1.6)	0.300	1.1 (0.8–1.5)	0.600
Medical practice	0.7 (0.5–0.9)	0.002	0.8 (0.6–1.0)	0.050
***Knowledge***				
Knowledge regarding influenza vaccine	4.1 (2.7–6.5)	<0.001	3.4 (2.1–5.7)	<0.001
Knowledge regarding COVID-19 vaccine	2.9 (1.5–5.9)	0.001	2.5 (1.2–5.6)	0.010
Knowledge regarding hepatitis B vaccine	3.2 (1.5–7.9)	0.002	2.1 (0.9–5.3)	0.070
***Source of information***				
Medical literature	1.5 (1.1–2.0)	0.003	1.8 (1.3–2.3)	<0.001
Official bulletins	1.1 (0.8–1.7)	0.500	0.8 (0.6–1.2)	0.300
Courses (from the faculty)	0.4 (0.2–0.7)	<0.001	0.4 (0.2–0.7)	0.001
General practitioners	1.2 (0.6–2.3)	0.600	1.8 (0.9–3.4)	0.060
Healthcare facility when I am doing the medical practice	1.2 (0.6–2.6)	0.700	1.0 (0.4–1.2)	0.900
Internet/social media/mass-media	0.5 (0.3–0.9)	0.009	0.6 (0.3–1.1)	0.060
Infectious disease specialist/epidemiologist	0.8 (0.4–1.3)	0.300	1.2 (0.7–2.0)	0.500
Colleagues	0.3 (0.03–1.4)	0.100	0.2 (0.02–0.8)	0.010
Did not receive any information on vaccines	0.2 (0.0–1.4)	0.080	0.2 (0.02–1.2)	0.050
***Practices***				
Annual influenza vaccination	39.8 (24.2–68)	<0.001	48.1 (31.6–73.6)	<0.001
***Previous vaccinations***				
COVID-19 vaccinated	3.7 (1.5–10.2)	0.001	4.0 (1.4–16)	0.005
Hepatitis B vaccinated	1.5 (1.1–1.9)	0.007	1.2 (0.9–5.3)	0.300

**Table 8:** Multivariable analysis of different exposures on the intention to get vaccinated against influenza in 2020–2021 and 2021–2022 seasons, Carol Davila University of Medicine and Pharmacy, Bucharest, Romania, 2021–2022.

	Vaccination Intention 2020–2021		Vaccination Intention 2021–2022	
	OR ^1^ (95% CI)	*p*-Value	OR ^1^ (95% CI)	*p*-Value
***Knowledge***				
Knowledge regarding influenza vaccine				
No	1		1	
Yes	2.5 (1.1–5.7)	0.030	3.2 (1.5–6.8)	0.003
Don’t know	0.6 (0.1–2.7)	0.500	0.6 (0.2–2.3)	0.500
Knowledge regarding COVID-19 vaccine				
No	1		1	
Yes	2.2 (0.6–8.1)	0.200	4.1 (1.1–15.0)	0.030
Don’t know	1.0 (0.1–12.6)	0.900	1.1 (0.1–13.2)	0.900
Knowledge regarding hepatitis B vaccine				
No	1		1	
Yes	0.5 (0.04–4.9)	0.500	0.3 (0.03–2.8)	0.300
Don’t know	not estimable		not estimable	
***Source of information***				
Medical literature	1		1	
Official bulletins	0.8 (0.4–1.9)	0.700	1.5 (0.8–3.1)	0.200
Courses (from faculty)	1.0 (0.4–2.5)	0.900	1.1 (0.5–2.5)	0.800
General Practitioners	3.9 (1.5–10.3)	0.005	2.4 (0.9–5.9)	0.070
Healthcare facility where I am practicing	0.9 (0.3–3.0)	0.900	1.1 (0.3–3.3)	0.900
Internet/social media/mass-media	1.2 (0.4–3.5)	0.700	1.3 (0.5–3.3)	0.600
Colleagues	0.5 (0.06–5.1)	0.600	0.3 (0.03–2.7)	0.300
Infectious disease specialist/epidemiologist	1.0 (0.4–2.6)	0.900	0.9 (0.3–2.2)	0.700
Did not receive any information on the vaccines	0.9 (0.08–11.9)	0.900	0.6 (0.05–7.9)	0.700
***Practices***				
Annual influenza vaccination				
No	1		1	
Yes	66.3 (31.4–140.0)	<0.001	44.5 (19.3–102.4)	<0.001
***Previous vaccinations***				
COVID-19 vaccinated				
No	1		1	
Yes	5.8 (0.7–46.3)	0.100	5.1 (1.1–23.6)	0.040
Hepatitis B vaccinated				
No	1		1	
Yes	0.9 (0.5–1.6)	0.700	0.8 (0.5–1.5)	0.600
Don’t know	0.8 (0.4–1.8)	0.600	0.5 (0.2–1.1)	0.070

^1^ Logistic Regression model adjusted for terms of faculty (Dental Medicine, Medicine); sex (male, female); year (IV, VI); period (<1 year, 1–3 years, >3 years); and age (continuous).

## Data Availability

The datasets generated and analyzed during the current study are available from the corresponding authors upon reasonable request.
